# Trends and Therapeutic Imperatives in Pulmonary Embolism Therapy

**DOI:** 10.1016/j.jscai.2026.105461

**Published:** 2026-06-02

**Authors:** Peter P. Monteleone, Paul S. Mueller

**Affiliations:** a Department of Internal Medicine, The University of Texas at Austin Dell School of Medicine, Austin, Texas; b Ascension Texas Cardiovascular, Austin, Texas

**Keywords:** pulmonary embolism, technologic imperatives, therapeutic imperatives

The PERT Consortium is a highly collaborative and forward-thinking international network dedicated to improving the care of patients with pulmonary embolism (PE) through multidisciplinary expertise, research, and education. In many ways, it is a model for multidisciplinary pragmatic care for other subspecialties as well as other pathology-focused societies. Perhaps its greatest value has been its support of clinical research in the field of PE. The consortium has sponsored multiple prospective analyses and lent its expertise to the development of leading clinical trials. In this issue of *JSCAI*, the research arm of the PERT Consortium presents what is arguably the most robust real-world description of current practices in the advanced therapies for PE.^1^

The field of advanced therapy for elevated-risk PE has grown exponentially. Over 2 decades, the devices available (both in numbers and mechanisms) and the number of operators experienced in endovascular treatment of PE have exploded. Although the disease state likely remains undertreated in many geographies, the spread of technology and expertise has been remarkable. With the arrival of the field’s first landmark clinical trial, HI-PEITHO, as well as publication of updated multisociety guidelines, these trends will undoubtedly accelerate.^2^^,^^3^

This National PERT Consortium registry analysis reports that 22.5% of patients in the registry were treated with catheter-based interventional therapies. Secemsky et al^4^ published a Medicare analysis and reported that the utilization of catheter-based interventional therapies increased from 5.92% in 2018 to 14.1% in 2023. This data discrepancy is likely due to the “enriched” population in the National PERT Consortium registry, ie, pulmonary embolism response team (PERT) centers that voluntarily contribute data to the registry likely represent champions of the field, with higher availability and utilization of these therapies. Furthermore, the patients in the registry likely do not comprise all the consecutively hospitalized patients with PE. Rather, the National PERT Consortium registry likely reports the presentation and outcomes of the patients whose data are submitted to the National PERT Consortium registry. In some centers, this reporting may represent only inpatients for whom the local PERT team is activated. Potentially, only patients in whom intervention is considered or even performed are included.

As the authors describe within this National PERT Consortium registry dataset, only 11.6% (n = 1342) of patients were categorized as low risk, whereas 73.7% (n = 8569) were categorized as intermediate, and 14.6% (n = 1701) were categorized as high risk. These data contrast with prior reports in which low-risk patients comprise approximately 40% to 50% of patients with PE.^5^^,^^6^ These discrepant findings also likely represent an “enriched” selection bias among National PERT Consortium registry patients. As a result, all summative and comparative results presented in this work need to be viewed with this bias in mind.

For example, the authors report that of the study cohort, 30-day and 1-year survival were numerically higher in patients undergoing surgical embolectomy than in patients undergoing inferior vena cava filter placement. Does that mean that in the real clinical world amidst all comers, the mortality risk of surgical embolectomy is lower than that of inferior vena cava filter placement? Of course not. Rather, the findings likely reflect the excellence of advanced surgical therapies at PERT centers and the broad risks associated with venous thromboembolism in patients for whom anticoagulation is contraindicated. This selection bias does not make the registry flawed or invalidate the reported results. Rather, it adds an important caveat to interpretation of the results.

Perhaps more importantly, these data should remind us that as we advance the field of PE therapy, we must remain thoughtful and grounded in objective and continuously improving data. As much as any other clinical pathology that might result in life-threatening physiologic compromise, PE is subject to an “imperative to treat.” When PE is diagnosed, and its pathophysiology is explained to a patient or a patient’s family (eg, “a blood clot from a leg vein has traveled to a lung artery”), a natural response is, “Can you take it out?” Embedded in this process is the drive to do something—to treat. Such a straightforward, quick, and dramatic “cure” to a life-threatening disease state typically does not exist for infectious diseases, chronic illnesses, or many other pathophysiologies. These factors drive the so-called “treatment imperative,” which is “the almost inexorable momentum toward an intervention that is experienced by physicians, patients, and family members alike.” In the setting of an abrupt, potentially life-ending illness like PE, in which quick decisions must be made, physicians may feel compelled to offer plausible yet unproven treatments to patients. Confronting possible death, it is hard for patients to decline such offers.^7^

As device technologies and therapies have improved, the desire to use them has also increased. The “technological imperative” is “giving the best care that is technically possible; the only legitimate and explicitly recognized constraint is the state of the art.”^8^ Procedures for the treatment of PE have become faster and safer. Clot extraction has become more robust. Blood return mechanisms with limited testing and evaluation became available, which presumably have limited blood loss and enhanced safety and availability of advanced thrombectomy techniques. Indeed, images of extracted clot sitting on procedural tables in neatly arranged figures matching the lung tree anatomy have popped up on social media, rousing wonder. Even patients request photos of “their clot.” This psychological drive to treat PE with advanced technologies has grown as the ability to do so has become more feasible, accepted, and available. In short, the “technological imperative” drives use. The growth of treatment options, proceduralist and equipment presence, and therapeutic applicability increase the drive to perform the procedure.

However, treatment and technological imperatives can unknowingly override clinical judgment regarding benefit and harms and shared decision-making. That is, the decision to intervene can be driven by a desire to intervene and technical expertise and availability of advanced technologies rather than by clinical benefit and appropriateness. Notably, treatment of PE is not alone regarding treatment and technological imperatives. These imperatives have been identified among various novel treatments, including in the cardiovascular space among implantable cardiac devices.^9^ However, as these more established therapeutic questions have been tackled, the PE community must similarly remain dedicated to certain fundamentals of clinical decision making. Our care must remain centered on patients’ values and healthcare goals. For this reason, we must continue to explore both clinical outcomes as well as quality-of-life outcomes in our patients (as many current and upcoming clinical trials will do).^2^^,^^10^^,^^11^ We must continuously seek to identify clinical situations in which these advanced therapies are nonbeneficial concurrently with our work to determine where these therapies work best. These are similar questions, but they are distinct.

Perhaps most importantly, we must avoid reflexively offering interventional therapies. As a field, one of the greatest steps toward this was taken in advance thanks to the immersion of multidisciplinary decision-making at the heart of the growth of PE therapies. This was a root success of the PERT Consortium responsible for the analysis presented in this issue of *JSCAI*; as a field, we should be grateful to this consortium for allowing the “decision to hammer” to be foundationally reviewed by more than just the individuals “with the hammer.” The analysis itself provides ample information for generating hypotheses and designing new studies that ultimately will result in continued improvement of the care of patients with PE.

The work presented by the PERT Consortium in this issue of *JSCAI* enhances our understanding of trends in PE patient presentation and currently offered therapies. It is a highly awaited update on real-world data from the group that perhaps more than any other has thoughtfully guided the hand of the exponential rise of advanced interventional therapies for PE. As we review these and other data and we pride ourselves as stewards of one of the fastest-growing fields in clinical care, it also provides an opportunity for us to step back and consider where we go next and why we choose to do so. As we derive the next decade in the field of PE therapy, we must remain committed to understanding the nuanced inertia behind our decisions. Clinical research and guideline directives should drive us, but cognizance of treatment and technological imperatives that we may not have intentionally planned will allow us to thoughtfully shape our next steps forward.

## CRediT authorship contribution statement

**Peter P. Monteleone:** Conceptualization, Writing – original draft, Writing – review & editing. **Paul S. Mueller:** Conceptualization, Writing – original draft, Writing – review & editing.

## Declaration of competing interest

Peter P. Monteleone serves on the scientific advisory boards of Boston Scientific and Medtronic. He provides consulting services to Boston Scientific, FlowPhysix, Medtronic, and Inquis Medical. Paul S. Mueller has no relevant disclosures.
